# Decreased survival in children inpatients with COVID-19 and antibiotic prescription

**DOI:** 10.1186/s12879-022-07516-x

**Published:** 2022-06-10

**Authors:** Efrén Murillo-Zamora, Xóchitl Trujillo, Miguel Huerta, Mónica Ríos-Silva, Agustin Lugo-Radillo, Oliver Mendoza-Cano

**Affiliations:** 1 Departamento de Epidemiología, Unidad de Medicina Familiar No. 19, Instituto Mexicano del Seguro Social, Av. Javier Mina 301, Col. Centro, C.P. 28000 Colima, Colima Mexico; 2 Facultad de Medicina, Universidad de Colima, Av. Universidad 333, Col. Las Víboras, C.P. 28040 Colima, Colima Mexico; 3 Centro Universitario de Investigaciones Biomédicas, Universidad de Colima, Av. 25 de julio 965, Col. Villas San Sebastián, C.P. 28045 Colima, Mexico; 4 Universidad de Colima - CONACyT, Centro Universitario de Investigaciones Biomédicas, Av. 25 de julio 965, Col. Villas San Sebastián, C.P. 28045 Colima, Mexico; 5 CONACYT - Facultad de Medicina y Cirugía, Universidad Autónoma Benito Juárez de Oaxaca, Oaxaca, Mexico; 6 Facultad de Ingeniería Civil, Universidad de Colima, km. 9 carretera Colima-Coquimatlán, C.P. 28400 Coquimatlán, Colima Mexico

**Keywords:** COVID-19, Child, Hospitalized, Anti-bacterial agents, Drug prescriptions, Fatal outcome

## Abstract

**Background:**

The empirical prescription of antibiotics to inpatients with Coronavirus Disease 2019 (COVID-19) is frequent despite uncommon bacterial coinfections. Current knowledge of the effect of antibiotics on the survival of hospitalized children with COVID-19 is limited.

**Objective:**

To characterize the survival experience of children with laboratory-positive COVID-19 in whom antibiotics were prescribed at hospital admission.

**Methods:**

A retrospective cohort study was conducted in Mexico, with children hospitalized due to COVID-19 from March 2020 to December 2021. Data from 1601 patients were analyzed using the Kaplan–Meier method and the log-rank test. We computed hazard ratios (HR) and 95% confidence intervals (CI) to evaluate the effect of the analyzed exposures on disease outcomes.

**Results:**

Antibiotics were prescribed to 13.2% ($n$ = 211) of enrolled children and a higher mortality rate [14.9 (95% CI 10.1–19.8) vs. 8.3 (95% CI 6.8–9.8)] per 1000 person-days, $p$ < 0.001) was found among them. At any given cut-off, survival functions were lower in antibiotic-positive inpatients ($p$ < 0.001). In the multiple model, antibiotic prescription was associated with a 50% increase in the risk of fatal outcome (HR = 1.50, 95% CI 1.01–2.22). A longer interval between illness onset and healthcare-seeking and pneumonia at hospital admission was associated with a poorer prognosis.

**Conclusions:**

Our results suggest that antibiotic prescription in children hospitalized due to COVID-19 is associated with decreased survival. If later replicated, these findings highlight the need for rational antibiotics in these patients.

## Background

The Coronavirus Disease 2019 (COVID-19) burden among children in Mexico has been high [1]. By mid-January 2022, and among children aged 9 years or younger, more than 85 thousand confirmed cases of COVID-19 had been registered, together with 7.3 thousand hospital admissions [2].

Latin-American countries had been hard-hit by the COVID-19 pandemic. In the region, the cumulative mortality rate (per 100 thousand people) observed in Mexico by the start of April 2022 (254) is only overcome by Peru (654), Brazil (314), Chile (302), Argentina (286), Colombia (278) and Paraguay (267) [3].

Current knowledge on managing adult or children patients with COVID-19 is insufficient [4]. Despite bacterial coinfections being infrequent and presented only in around 8% of patients [5], the empirical use of antibiotics in patients with COVID-19 has been widely documented since the start of the epidemic in the city of Wuhan, China [6].

The proportion of COVID-19 Chinese patients receiving antibiotics during hospital stay was around 50% [7, 8]. In general, higher rates have been registered in the United States and European countries, above 70% [9, 10].

Antibiotic prescription rates as high as 86% have been documented in hospitalized children [11]. This is particularly concerning since a poorer survival has been reported among COVID-19 adult inpatients receiving antibiotics [10]; moreover, to the best of our knowledge, there are not hitherto published studies evaluating in-hospital outcomes in children patients receiving antibiotics. This study aimed to characterize the survival experience of children hospitalized due to a laboratory-positive COVID-19 result in whom antibiotics were prescribed.

## Methods

### Study design and setting

We conducted a nationwide retrospective cohort study in Mexico from November 2021 to January 2022. Children that were hospitalized due to laboratory-confirmed (reverse-transcription polymerase chain reaction, RT-PCR) COVID-19 were potentially eligible. They were identified from the nominal records found in a national and normative system for epidemiological surveillance of respiratory viruses, which belongs to the Mexican Institute of Social Security (*IMSS*, the Spanish acronym). IMSS is part of the public healthcare system. It provides medical assistance, social protection, and integral services to its users through more than 6.5 thousand medical units (350 and 36 of them being secondary and tertiary care hospitals, respectively) located across Mexico.

### Study population

According to normative standards, RT-PCR testing is performed in all suspected cases of COVID-19 requiring hospital admission [12]. Hospitalized children aged 9 years or younger, with the onset of symptoms of COVID-19 from March 2020 to December 2021 and with conclusive test results, were eligible. Individuals with missing clinical or epidemiologic data of interest were excluded.

### Data collection

Data of interest were retrieved from the audited surveillance system, which primary data sources were the medical records of enrolled patients and death certificates, if applicable. Analyzed information included demographic characteristics (sex, age), personal history of noncommunicable diseases (no/yes: obesity, type 1 diabetes mellitus, asthma, chronic kidney disease, immunosuppression or cardiovascular disease), administration of antibiotics (any; no/yes) at hospital admission, clinical manifestations (no/yes: fever or chills, cough, shortness of breath, and tachypnea) and pneumonia-related radiographic findings (no/yes: ground glass patterns in X-ray or computed tomography scanning). Hospitalized children with pneumonia at admission were those with both clinical manifestations and radiographic findings of this abnormality [13].

Date of healthcare-seeking and dates of hospital admission and discharge (and the causes of hospital discharge [recovery/death], were also extracted from the audited database. The interval (days) elapsed between the symptoms onset and the date of healthcare-seeking were computed.

We used the date of symptom onset as an approximation for the SARS-COV-2 variant causing the infection and were categorized as March 2020–April 2021 or May 2021–December 2021, when the dominant variants were the ancestral and Delta (B.1.617.2), respectively [14].

### Outcome

The primary outcome was the cause [recovery or death (due to any immediate cause)] of hospital discharge of children hospitalized due to COVID-19.

### Laboratory methods

Clinical specimens (deep nasal swabs) were analyzed (SuperScript™ III Platinum™ One-Step qRT-PCR Kits) at four specialized regional laboratories integrated into the IMSS network for epidemiologic surveillance. A broader description of the laboratory methods has already been published elsewhere [15].

### Statistical analysis

We computed summary statistics and the significance level (α) at 5%. The Kaplan–Meier method calculated survival functions and 95% confidence intervals (CI). The log-rank test was used to compare the survival distributions of the study groups. The effect of the prescription of antibiotics (any) was evaluated through hazard ratios (HR), and 95% CI was computed using a multivariate Cox proportional hazard regression model. All analyses were conducted using Stata version 16.0 (StataCorp; College Station, TX, USA).

## Results

**Fig. 1:**
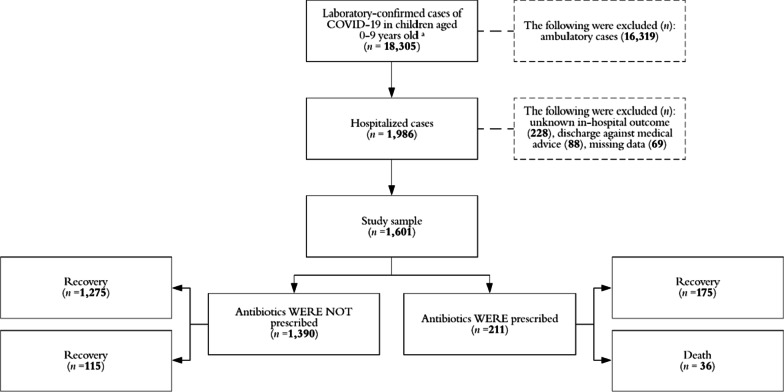
Study profile, Mexico 2020–2021. ^a^Cases registered, from March 2020 to December 2021, in a national and normative system for the epidemiological surveillance of respiratory viruses, which belongs to the Mexican Institute of Social Security

Data from 1601 patients were analyzed for a total follow-up of 16,238 person-days. Figure 1 shows the study profile. Antibiotic prescription was identified in 13.2% ($n$ = 211/1601; 95% CI 11.6–14.9%) of analyzed inpatients and the overall frequency, according to disease outcome, was 12.1% (95% CI 10.5–13.9%) vs. 23.8% (95% CI 17.0–30.6%) in non-fatal and fatal cases respectively ($p$ < 0.001). Cephalosporins were the most commonly prescribed antibiotics.

The overall rate of a fatal in-hospital outcome was 9.3 ($n$ = 151/16,238; 95% 7.8–10.8) per 1000 person-days. The mortality rate was higher among antibiotic-positive inpatients [14.9 (95% CI 10.1–19.8) vs. 8.3 (95% CI 6.8–9.8) per 1000 person-days, $p$ < 0.001]. Table 1 summarizes the characteristics of participants for selected variables.

**Table 1:** Characteristics of the analyzed children with COVID-19 according to in-hospital outcome, Mexico 2020–2021

Characteristic	Overall		In-hospital outcome				$p$	Follow-up (person-days)
			Recovery		Death			
	$n$ = 1601		$n$ = 1450		$n$ = 151			
Sex								
Girl	54 (40.8)		585 (89.4)		69 (10.6)		0.203	6750
Boy	947 (59.2)		865 (91.3)		82 (8.7)			9488
Age (years)^a^	1.7 (0.3–4.8)		1.7 (0.2–4.8)		1.5 (0.3–4.6)		0.925	16,238
Age group (years)								
< 1	653 (40.8)		587 (89.9)		66 (10.1)		0.694	7168
1–4	576 (36.0)		526 (91.3)		50 (8.7)			5622
5–9	372 (23.2)		337 (90.6)		35 (9.4)			3448
Date of symptoms onset								
March 2020–April 2021	901 (56.3)		794 (88.1)		107 (11.9)		< 0.001	10,910
May 2021–December 2021	700 (43.7)		656 (93.7)		44 (6.3)			5328
Days from symptoms onset to healthcare-seeking^a^	1 (0–4)		1 (0–4)		1 (0–4)		0.906	16,238
Antibiotic (any) was prescribed at hospital admission								
No	1390 (86.8)		1275 (91.7)		115 (8.3)		< 0.001	13,825
Yes	211 (13.2)		175 (82.9)		36 (17.1)			2413
Prescribed antibiotic^b^								
Cephalosporin	65 (30.8)		55 (84.6)		10 (15.4)		0.206	648
Penicillin	22 (10.4)		22 (100)		0 (0)			278
Macrolide	12 (5.7)		9 (75.0)		3 (25.0)			77
Aminoglycoside	11 (5.2)		10 (90.9)		1 (9.1)			90
Glycopeptide	11 (5.2)		7 (63.6)		4 (36.4)			154
Carbapenem	4 (1.9)		1 (25.0)		3 (75.0)			24
Unspecified	86 (40.8)		71 (82.6)		15 (17.4)			1142
Length of hospital stay (days)^a^	6 (3–14)		6 (3–14)		7 (3–17)		0.095	16,238
Personal history of								
Obesity (yes)	25 (1.6)		22 (88.0)		3 (12.0)		0.658	261
Asthma (yes)	62 (3.9)		57 (91.9)		5 (8.1)		0.707	611
T1DM (yes)	21 (1.3)		19 (90.5)		2 (9.5)		0.988	223
Cardiovascular disease (yes)	57 (3.5)		44 (77.2)		12 (21.1)		0.002	593
CKD (yes)	9 (0.6)		7 (77.8)		2 (22.2)		0.188	101
Immunosuppression (yes)	99 (6.2)		83 (83.8)		14 (14.1)		0.082	1004

(1) $p$-value from chi-squared or Mann–Whitney U test is presented as corresponding; (2) Absolute ($n$) and relative (%) frequencies are presented, except if median and interquartile ranges are specified; (3) Percentages of death by row are shown

*COVID-19* Coronavirus disease 2019; *T1DM* type 1 diabetes mellitus; *CKD* chronic kidney disease

^a^Median and interquartile ranges

^b^Among 211 hospitalized children in whom antibiotics were prescribed

**Fig. 2:**
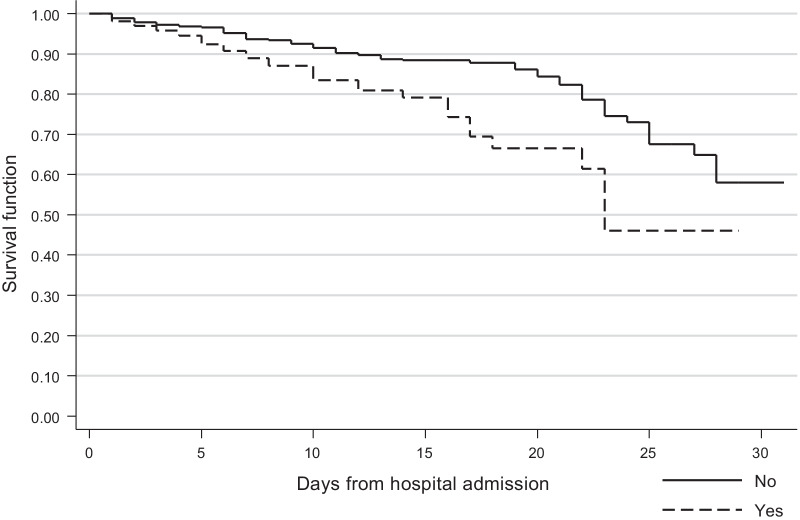
Survival function of hospitalized children with COVID-19 according to the prescription of antibiotics (any) at hospital admission, Mexico 2020–2021. Log-rank test, $p$ = 0.001

As presented in Fig. 2, survival rates were lower among patients prescribed antibiotics, specifically on day 15 of hospital admission and later (log-rank test, p = 0.001). The specifics survival rates (antibiotics were prescribed, yes vs. no), according to the number of days elapsed since hospital entry, were as follows: 1 day, 98.0% (95% CI 94.8–99.3%) vs. 98.8% (95% CI 98.0–99.3%); 3 days, 96.9% (95% CI 93.2–98.6%) vs. 97.7% (95% CI 96.7–98.4%); 7 days, 91.0% (95% CI 85.6–94.5%) vs. 95.2% (95% CI 93.8–96.4%); 15 days, 82.3% (95% CI 74.9–87.7%) vs. 90.1% (95% CI 87.9–91–9%); 21 days, 77.6% (95% CI 69.6–83.8%) vs. 88.8% (95% CI 86.4–90.6%); and 30 days, 73.4% (95% CI 64.9–80.2%) vs. 86.5% (95% CI 83.8–88.7%).

In the multiple regression model (Table 2), and compared with children who did not receive antibiotics, hospitalized children receiving any of these drugs had a 50% increased risk of fatal outcome (HR = 1.50, 95% CI 1.01–2.22). We also documented a poorer in-hospital outcome in patients with more days elapsed between illness onset and healthcare-seeking (per each additional day: HR = 1.05, 95% CI 1.01–1.10), as well as a nearly twofold increase in the risk of death among children with pneumonia at hospital entry (HR = 1.94, IC 95% 1.37–2.77).

**Table 2:** Predictors of survival of hospitalized children with COVID-19, Mexico 2020–2021

Characteristic	HR (95% CI),$p$					
	Bivariate analysis			Multiple analysis		
Sex						
Girl	1.00			1.00		
Boy	0.84 (0.61–1.16)		0.301	0.82 (0.59–1.13)		0.227
Age group (years)						
< 1	1.00			1.00		
1–4	0.96 (0.67–1.39)		0.836	0.97 (0.67–1.41)		0.883
5–9	1.11 (0.74–1.67)		0.617	1.13 (0.75–1.71)		0.556
Date of symptoms onset						
March 2020–April 2021	1.00			1.00		
May 2021–December 2021	0.86 (0.60–1.23)		0.401	0.88 (0.61–1.27)		0.504
Days from symptoms onset to healthcare-seeking	1.06 (1.01–1.11)		0.015	1.05 (1.01–1.10)		0.040
Antibiotic (any) was prescribed at hospital admission						
No	1.00			1.00		
Yes	1.82 (1.25–2.66)		0.002	1.50 (1.01–2.22)		0.043
Pneumonia diagnosis at hospital admission						
No	1.00			1.00		
Yes	2.15 (1.53–3.03)		< 0.001	1.94 (1.37–2.77)		< 0.001
Cardiovascular disease						
No						
Yes	2.04 (0.94–4.44)		0.073	1.90 (0.87–4.14)		0.106

(1) Generalized linear regression models were used to obtain the presented estimates; (2) HR and 95% CI from the multiple analysis were adjusted by the variables presented in the table

*COVID-19* Coronavirus disease 2019; *HR* hazard ratio; *CI* confidence interval

## Discussion

We characterized the survival experience of a large subset of children inpatients with laboratory-confirmed COVID-19. Our findings suggest that prescribing antibiotics to pediatric COVID-19 patients is associated with a poorer in-hospital prognosis. However, given the limitations of an observational study, the results must be carefully considered.

Published data regarding the antibiotic prescription rates in hospitalizes are scarce, and the estimates heterogeneous. In our study, these drugs were prescribed to around 13% of enrolled inpatients. This frequency is almost a half (24.5%, p < 0.001) of the rate documented by a previously published Latin-American multicenter study where most of the analyzed patients were from Peru and Costa Rica [16]. Our rate is also lower than the computed by a metanalysis where 154 studies were analyzed (38.5%, 95% 26.3–52.3%; p < 0.001) [9].

We hypothesize that two factors may be determined, at least partially, by the low rates of antibiotic prescription documented in our study. The first is that all hospitals from where patients were recruited were public settings belonging to IMSS. For-profit hospitals have been associated with a higher risk of receiving these drugs [17]. The second factor is that IMSS developed early (April 2020) protocols for the attention of COVID-19 patients [18].

The main factors determining the start of antibiotics seem to be increased inflammatory markers, and any infiltrate on an x-ray image [19]. In our study sample, children inpatients with pneumonia were more likely to receive antibacterial drugs at admission (31.1% vs. 9.1%, p < 0.001).

The empirical prescription of antibiotics in COVID-19 patients does not reduce the risk for severe symptoms or death [20]. The clinical usefulness of macrolides, for their anti-inflammatory properties, is also questionable [21, 22].

The overuse of antibiotics in patients with COVID-19, especially combinations of broad-spectrum antibiotics, has become a significant concern [23]. Fighting the threat of antibiotic resistance is a public health priority as crucial to limiting the spread of SARS-COV-2, especially in children with a high respiratory infection rate [24].

The potential limitations of our study must be cited. First, data regarding the prescription of antibiotics were collected as a dichotomous variable, and other clinical and epidemiological relevant information (such as length of administration) was omitted. We also documented that in around 40% of inpatients, the precisely prescribed antibiotic was not registered. Second, we did not evaluate intermediate outcomes such as the multisystem inflammatory syndrome temporally related to COVID-19 [25], which might impact antibiotic prescription rates. Third, the initial symptoms of COVID-19 may be unspecific [26]. Therefore, we recommend carefully considering our slight but significant increased risk of death among children with more days from symptom onset to healthcare seeking. Finally, we used anonymized data, and we were unable to identify what patients received medical care in secondary and tertiary care hospitals. The latter may have had any effect on the observed estimates. However, above 90% of units from which patients were recruited were secondary care hospitals.

## Conclusions

Our findings suggest that antibiotic prescription in children inpatients with COVID-19 is associated with an increased risk for fatal outcome. If later replicated in other populations, our results highlight the major relevance of limiting the empirical administration of anti-bacterial drugs in these patients.

## Data Availability

All data generated or analyzed during this study are included within this published article.
